# Genomic characteristics of trastuzumab-resistant Her2-positive metastatic breast cancer

**DOI:** 10.1007/s00432-017-2358-x

**Published:** 2017-02-28

**Authors:** Mateus de Oliveira Taveira, Sheida Nabavi, Yuker Wang, Peter Tonellato, Francisco J. Esteva, Lewis C. Cantley, Gerburg M. Wulf

**Affiliations:** 1 Department of Medicine, Beth Israel Deaconess Medical Center, Boston, MA USA; 2 Harvard Medical School, Boston, MA USA; 3 University of Connecticut, Storrs, CT USA; 4 Affymetrix, San Francisco, CA USA; 5 Vitruvian Networks, San Francisco, CA USA; 6 MD Anderson, Houston, TX USA; 7 Perlmutter Cancer Center, NYU Langone Medical Center, New York, NY USA; 8 Weill Cornell Medical College, New York, NY USA

**Keywords:** Her2, Trastuzumab, Everolimus, Metastatic breast cancer, Phosphoinositol 3-kinase, mTOR

## Abstract

**Purpose:**

Resistance to trastuzumab therapy is linked to phosphoinositol 3-kinase (PI3K) pathway activation. One key downstream effector and regulator of this pathway is the mechanistic target of rapamycin (mTOR). In 2011, a phase I/II study evaluated the combination of trastuzumab and everolimus (a mTOR inhibitor) for treatment of Her2-positive metastatic breast cancer (MBC) for patients who had progressed on trastuzumab-based therapy.

**Methods:**

We retrospectively analyzed GeneChip microarray data from 22 of 47 patients included in the study.

**Results:**

Using an unbiased approach, we found that mutations in *BRAF, EGFR* and *KIT* are significantly more common in this heavily treated population when compared with the cohort of invasive breast carcinoma patients in The Cancer Genome Atlas (TCGA). Furthermore, 10 out of 22 patients had *PIK3CA* mutations (45.4%) but *PI3KCA* status was not predictive of PFS in our cohort. Finally, the use of OncoScan^tm^ has allowed us to detected mutations in five genes that have not been shown to be mutated in TCGA subset of Her-2 overexpressing breast cancer: *CTNNB1, HRAS, KRAS, NF2* and *SMARCB1*.

**Conclusion:**

Mutational burden in heavily treated trastuzumab-resistant Her2-positive metastatic breast cancer is highly variable and not directly correlated with outcome. Activation of the MAPK/ERK pathway through mutations in *EGFR, BRAF* or *KIT* may mediate resistance to trastuzumab.

## Introduction

Breast cancer (BC) is the most prevalent non-skin cancer in women, accounting for ~40,000 deaths per year in the USA (Siegel et al. 2012). About one quarter of patients with BC overexpress Her2, which is associated with decreased overall survival (OS) (Slamon et al. 1987). The majority of patients with Her2-positive disease receive trastuzumab as part of their treatment. This monoclonal antibody targets the extracellular domain of the HER2 receptor and has significantly increased OS for this subset of patients (Slamon et al. 2001). Unfortunately, not all patients respond to trastuzumab-based therapies and some develop secondary resistance after disease remission. The precise mechanisms behind trastuzumab resistance are not fully understood, but some have implicated activation of the phosphoinositol 3-kinase (PI3K) pathway (Berns et al. 2007). Activation of PI3K pathway through *PIK3CA* mutations or PTEN loss would lead to cell growth through mTOR-mediated signaling, effectively rendering proliferating signals from epidermal growth factor receptors, such as HER2, redundant (Nagata et al. 2004). Multiple phase I/II trials were done to evaluate the safety and efficacy of using everolimus—a mTOR inhibitor—in patients who had progressed on trastuzumab-based therapies (André et al. 2014, 2016; Hurvitz et al. 2015; Morrow et al. 2011a). In the present study, we have retrospectively analyzed the genomic characteristics of patients included in one of such studies, whose results were published in the Journal of Clinical Oncology in 2011 (Morrow et al. 2011a).

## Results

### Mutations in trastuzumab-resistant Her2-positive metastatic breast cancer

Archival tumor, pre-dating the exposure to trastuzumab and everolimus, was used for DNA extraction. After quality control, 22 samples were submitted to DNA microarray panel (Affymetrix Oncoscan^™^), a genomic screening tool based on molecular inversion probe (MIP) technology for identifying copy number alterations, loss of heterozygosity (LOH), and somatic mutations (Wang et al. 2005, 2007, 2009). We identified 42 different mutations in 26 genes (Table 1). The most frequently mutated gene was *PIK3CA* (ten patients—45.4%) followed by *BRAF, EGFR* and *KIT* (two patients each—13.6%) (Table 2).

**Table 1:** List of mutations found in tumor DNA using Affymetrix OncoScan^™^ GeneChip

Gene: mutation	# of patients
ABL1: MUT = Y253H	1
APC: MUT = Q1294X	1
APC: MUT = R1114X	1
ATM: MUT = Q2442P	1
BRAF: MUT = G469E	1
BRAF: MUT = I326T	2
BRCA1: MUT = G778C	2
BRCA1: MUT = W372X	1
CSF1R: MUT = Y969H	1
CTNNB1: MUT = D32N	1
CTNNB1: MUT = S45P	1
EGFR: MUT = R108K	2
EGFR: MUT = R677H	1
ERBB2: MUT = L755S	1
FBXW7: MUT = R393X	1
HRAS: MUT = Q61P	1
KIT: MUT = E839K	2
KIT: MUT = V825A	1
KRAS: MUT = Q61K	2
MAP2K4: MUT = S184L	1
MET: MUT = 982 complex variant	1
MET: MUT = R988C	1
MSH2: MUT = R711X	1
NF1: MUT = R304X	1
NF2: MUT = Q362X	2
NF2: MUT = R262X	1
PIK3CA: MUT = C901F	2
PIK3CA: MUT = E542K	2
PIK3CA: MUT = E545K	4
PIK3CA: MUT = H1047R	5
PIK3CA: MUT = R108H	2
PIK3CA: MUT = R38H	1
PTEN: MUT = C165	1
SMAD4: MUT = E330A	1
SMAD4: MUT = R445X	1
SMARCB1: MUT = R158X	1
SMARCB1: MUT = Y47X	1
SMO: MUT = W535L	2
WT1: MUT = F154S	1
WT1: MUT = R301X	1

**Table 2:** Prevalence of mutations in each gene represented in our cohort ranked according to the most mutated genes in TCGA cohort

	TCGA: Her2− only (*n* = 985)	TCGA: Her2 + only (*n* = 120)	JCO 2011 (*n* = 22)
*PIK3CA*	303 (30.8%)	37 (30.8%)	10 (45.4%)
*TP53*	290 (29.4%)	56 (46.7%)	n/a
*PTEN*	50 (5.1%)	4 (3.3%)	1 (4.5%)
*MAP2K4*	32 (3.2%)	2 (1.7%)	1 (4.5%)
*NF1*	29 (2.9%)	4 (3.3%)	1 (4.5%)
*BRCA1*	23 (2.3%)	1 (0.8%)	3 (13.6%)
*ATM*	22 (2.2%)	3 (2.5%)	1 (4.5%)
*ERBB2*	21 (2.1%)	4 (3.3%)	1 (4.5%)
*APC*	14 (1.4%)	2 (1.7%)	2 (9.0%)
*FBXW7*	14 (1.4%)	1 (0.8%)	1 (4.5%)
*ABL1*	7 (0.7%)	2 (1.7%)	1 (4.5%)
*EGFR*	7 (0.7%)	1 (0.8%)	3 (13.6%)
*KIT*	7 (0.7%)	1 (0.8%)	3 (13.6%)
*KRAS*	7 (0.7%)	0 (0.0%)	2 (9.0%)
*MET*	7 (0.7%)	2 (1.7%)	2 (9.0%)
*MSH2*	6 (0.6%)	2 (1.7%)	1 (4.5%)
*SMAD4*	6 (0.6%)	2 (1.7%)	2 (9.0%)
*NF2*	4 (0.4%)	0 (0.0%)	2 (9.0%)
*SMARCB1*	4 (0.4%)	0 (0.0%)	2 (9.0%)
*SMO*	4 (0.4%)	1 (0.8%)	2 (9.0%)
*HRAS*	3 (0.3%)	0 (0.0%)	1 (4.5%)
*BRAF*	2 (0.2%)	3 (2.5%)	3 (13.6%)
*CSF1R*	2 (0.2%)	2 (1.7%)	1 (4.5%)
*CTNNB1*	2 (0.2%)	0 (0.0%)	2 (9.0%)
*WT1*	2 (0.2%)	1 (0.8%)	2 (9.0%)

Data presented as numbers of patients with mutated gene and percentage of patients affected in that cohort

The Oncoscan™ method identified fewer mutations in *TP53* than would be expected (Nik-Zainal et al. 2016; Ciriello et al. 2015). This is likely due to the fact that the chip-based assay did not capture the range of different *TP53* mutations seen in breast cancer. For example, in the BC TCGA cohort there are 346 patients with 201 different *TP53* mutations (Ciriello et al. 2015). Our probe panel included 34 different probes targeting *TP53* mutations (Table 3). Only 21 of those are represented in the group of 201 mutations described in TCGA in BC (Ciriello et al. 2015). Hence, it is not surprising that we only found a single mutation (a mutation on codon 220 that leads to a change from tyrosine to cysteine), already described in BC and present in only six of the 346 patients with mutated *TP53* in TCGA BC cohort. Because of this methodological constraint, we excluded *TP53* from analysis of our cohort. Of note, *TP53* was differentially mutated in Her2-positive (*n* = 120) versus Her2-negative (*n* = 985) tumors in BC TCGA with 46.7% of Her2-positive tumors carrying a *TP53* mutation versus 29.4% of Her2-negative tumors (Table 4a, *p* = 0.0002).

**Table 3:** Tags used to screen for *TP53* mutations using Affymetrix OncoScan^™^

Tag number	Assay external id
tag100124	TP53_pR342X_c1024C_T
tag100067	TP53_pE336X_c1006G_T
tag110908	TP53_pQ331X_c991C_T
tag110798	TP53_p_c920_minus_1G_A
tag100123	TP53_pR306X_c916C_T
tag100066	TP53_pE298X_c892G_T
tag100122	TP53_pE285K_c853G_A
tag100169	TP53_pR273H_c818G_A
tag100168	TP53_pR273C_c817C_T
tag100544	TP53_pG266E_c797G_A
tag110665	TP53_p_c782_plus_1G_T
tag100167	TP53_pR249S_c747G_T
amp904	TP53_pR248Q_c743G_A
amp905	TP53_pR248W_c742C_T
amp894	TP53_pG245S_c733G_A
tag100959	TP53_pY236C_c707A_G
tag100120	TP53_p_c672_plus_1G_A
amp913	TP53_pY220C_c659A_G
tag101899	TP53_pR213X_c637C_T
amp902	TP53_pR196X_c586C_T
tag100165	TP53_pH193R_c578A_G
tag100069	TP53_pH179Q_c537T_G
tag100070	TP53_pH179R_c536A_G
tag100065	TP53_pC176F_c527G_T
tag100072	TP53_pR175H_c524G_A
tag100076	TP53_pY163C_c488A_G
amp888	TP53_pA159V_c476C_T
tag100071	TP53_pR158H_c473G_A
tag100075	TP53_pV157F_c469G_T
amp889	TP53_pC135F_c404G_T
tag100166	TP53_pK132Q_c394A_C
tag100164	TP53_p_c376_minus_1G_A
tag110632	TP53_pC124R_c370T_C
tag110809	TP53_pF113C_c338T_G

**Table 4:** Comparison between number of patients with mutations and without mutations in each cohort using Fischer’s exact test

a. TCGA Her2- vs TCGA Her2+		b. TCGA Her2 + vs JCO 2011		c. JCO 2011 versus TCGA	
Gene	*P* value	Gene	*P* value	Gene	*P* value
ABL1	0.3386	ABL1	0.3989	ABL1	0.1625
APC	0.6896	APC	0.1134	APC	0.0454
ATM	0.7470	ATM	0.4940	ATM	0.4018
BRAF	0.0106	BRAF	0.0477	BRAF	<**0.0001**
BRCA1	0.5041	BRCA1	0.0118	BRCA1	0.0170
CSF1R	0.0606	CSF1R	0.3989	CSF1R	0.0642
CTNNB1	1.0000	CTNNB1	0.0231	CTNNB1	0.0027
EGFR	0.6026	EGFR	0.0118	EGFR	**0.0010**
ERBB2	0.3388	ERBB2	0.5747	ERBB2	0.3881
FBXW7	1.0000	FBXW7	0.2868	FBXW7	0.2837
HRAS	1.0000	HRAS	0.1549	HRAS	0.0847
KIT	0.6026	KIT	0.0118	KIT	**0.0010**
KRAS	1.0000	KRAS	0.0231	KRAS	0.0150
MAP2K4	0.5721	MAP2K4	0.3989	MAP2K4	0.5233
MET	0.2548	MET	0.1134	MET	0.0150
MSH2	0.2125	MSH2	0.3989	MSH2	0.1437
NF1	0.7754	NF1	0.5747	NF1	0.4896
NF2	1.0000	NF2	0.0231	NF2	0.0065
PIK3CA	1.0000	PIK3CA	0.2194	PIK3CA	0.1631
PTEN	0.5062	PTEN	0.5747	PTEN	1.0000
SMAD4	0.2125	SMAD4	0.1134	SMAD4	0.0118
SMARCB1	1.0000	SMARCB1	0.0231	SMARCB1	0.0065
SMO	0.4378	SMO	0.0626	SMO	0.0065
TP53	**0.0002**	TP53	n/a	TP53	n/a
WT1	0.2919	WT1	0.0626	WT1	0.0027

*P* < 0.0020 defined as significant (in bold) based on Bonferroni correction for multiple comparisons. (a) Comparison between TCGA Her2-negative BC (*n* = 985) and TCGA Her2-positive BC (*n* = 120); (b) comparison between TCGA Her2-positive BC (*n* = 120) and this study Her2-positive trastuzumab-resistant MBC cohort (*n* = 22); (c) comparison between this study Her2-positive trastuzumab-resistant MBC cohort (*n* = 22) and all TCGA BC (*n* = 1105)

**Fig. 1:**
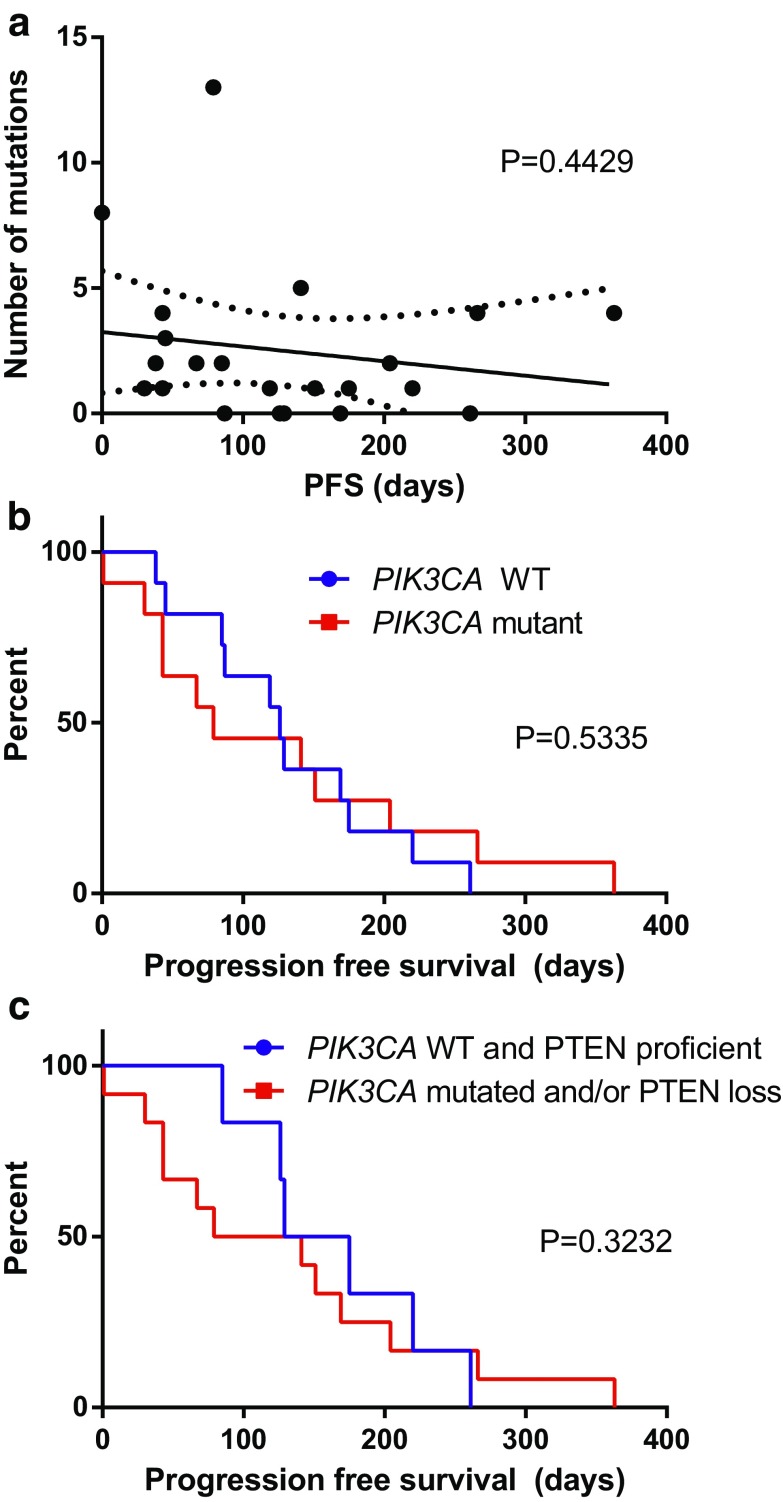
**a** Progression-free survival (PFS) does not correlate with total number of mutations per patient. *Continuous line* represents the linear regression. *Dotted line* is the 95% confidence interval. *P* = 0.4429. **b** PFS does not differ according to *PIK3CA* status (wild type versus mutated). *P* = 0.5335. **c** PFS does not differ according to PI3K pathway activation status, defined as *PIK3CA* mutation and/or PTEN loss. *P* = 0.3232

The total number of mutations was variable (Table 5) between patients, and OncoScan^™^ did not detect any mutations in 6/22 patients. Mutational burden did not correlate with progression-free survival (PFS) (*R* square = 0.0297, *P* = 0.4429) (Fig. 1a).

**Table 5:**
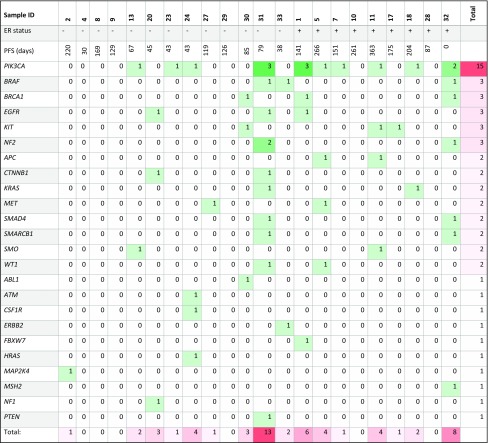
Mutational landscape of all samples included in the analysis

*PFS* progression-free survival; *ER* estrogen receptor; + = positive, − = negative; numbers represent the number of mutations in gene, 0 means no mutations were found in that gene in that patient (i.e. wild type)

Aside from TP53, which cannot be compared because of the methodological limitations mentioned above, the mutational pattern found in our patient population (*n* = 22), who had trastuzumab-resistant MBC, was not significantly different (defined as *P* < 0.0020, see Methods) from Her2-positive samples in TCGA (Table 5b). Of note, five genes mutated in our cohort—*CTNNB1, HRAS, KRAS, NF2, SMARCB1*—had not been previously observed in Her2-positive tumors in TCGA.

Finally, we compared our cohort with all TCGA breast cancer samples (*n* = 1105) (Table 5c). Three genes were found to be more commonly mutated amongst our patients than in TCGA: *BRAF* (13.6 vs 0.2%, *P* = 0.0001), *EGFR* (13.6 vs 0.7%, *P* = 0.0010) and *KIT* (13.6 vs 0.7%, *P* = 0.0010).

### Mutations in *PIK3CA* did not show prognostic value in our cohort

In our cohort, we found six different *PIK3CA* mutations, occurring in 10/22 patients. The difference in prevalence in our cohort (45.4%) and TCGA (30.8%) did not reach statistical significance (*P* = 0.1631). *PIK3CA* is the most frequently mutated gene in our cohort and second only to *TP53* in TCGA. Out of the ten patients in our cohort, two patients had three mutations in *PIK3CA*, one patient had two mutations and the remaining seven had one mutation each (Table 5). Not surprisingly, the most common mutation found was the mutation H1047R, found in five patients. It localizes to the highly conserved kinase domain and results in enhanced downstream signaling (Kang et al. 2005). E545K and E542K mutations were found in four and two patients respectively. Both localize to the helical domain and also lead to enhanced downstream signaling (Kang et al. 2005). In our cohort, two patients had a C901F mutation, one of them having two other concomitant mutations in *PIK3CA* (E545K, R108H). The C901F mutation localizes to the kinase domain and results in a cysteine to phenylalanine change. It has been described in endometrial carcinoma (Garcia-Dios et al. 2013), but it is rare in BC. In TCGA patients with BC it was found only in one patient, concurrent with an E542K mutation. The two other mutations in *PIK3CA* found in our cohort, R108H and R38H, were present in two and one patients, respectively. R38H localizes to the p85-binding domain of PI3K and R108H is a mutation that changes an arginine into a histidine in the amino acid immediately following the p85-binding domain. Both of these mutations have been described in endometrial carcinoma (Samuels et al. 2004) but are rare in BC. R38H is not present in BC TCGA cohort and R108H is found in only one patient in conjunction with the more common H1047R mutation.

Based on previous studies that suggested that activation of the PI3K pathway in Her2-positive tumors (through either *PIK3CA* mutations or *PTEN* loss) is associated with a worse prognosis (Andre et al. 2016), we analyzed whether *PIK3CA* mutations in our patients carried any prognostic value. From the 22 patients included, 12 had wild-type *PIK3CA* and 10 had *PIK3CA* mutations. Median PFS on treatment with trastuzumab and everolimus was 126.0 and 79.0 days, respectively, but PFS curves did not differ significantly between the two groups (*P* = 0.5353) (Fig. 1b). We also grouped patients with *PIK3CA* mutation and/or PTEN loss, both of which lead to PI3K activation, and compared them with patients without neither *PIK3CA* mutation nor PTEN loss. We excluded four patients from this analysis since PTEN status was not available. This comparison also yielded a non-significant result: median PFS of 152.0 and 110.0 days, respectively (*P* = 0.3232) (Fig. 1c).

### Influence of estrogen receptor (ER) status on mutations in Her2-positive BC

Finally, we examined whether there were mutations that were exclusively present in Her2-positive ER-positive versus Her2-positive ER-negative tumors in our cohort as the presence or absence of ER may have an impact on therapeutic decisions and efficacy (Schettini et al. 2016). In our patients, *ABL1, ATM, CSF1R, CTNNB1, ERBB2, HRAS, MAP2K4, NF1* and *PTEN* mutations were found only on Her2-positive ER-negative BC (*n* = 13) while *APC, FBXW7* and *MSH2* mutations were solely found on Her2-positive ER-positive samples (*n* = 9).

## Discussion

Our study describes the genetic background of breast tumors from a highly selected population of patients with trastuzumab-resistant Her2-positive metastatic breast cancers. The advent of trastuzumab and other therapies that target HER2/neu has changed the clinical course for patients diagnosed with this subset of BC (Balduzzi et al. 2014). Even more effective therapies using trastuzumab emtansine (T-DM1), an antibody–drug conjugate, are now available (Verma et al. 2012). Unfortunately, resistance to trastuzumab and other therapies that target the HER2/neu pathway still occurs and some patients do not benefit from trastuzumab-based drug regimen. That prompted phase I/II clinical trials that evaluated the benefit of adding everolimus to trastuzumab-based therapies after progression of disease while on trastuzumab (Morrow et al. 2011b). The combination of everolimus and trastuzumab led to partial responses (PR) in 7/47 patients (15%) and persistent stable disease (PSD) in 9/47 patients (19%), which amounts to a clinical benefit rate (CBR) of 34% (Morrow et al. 2011b).

In BOLERO-3—a randomized, double-blind, placebo-controlled phase 3 trial that evaluated adding everolimus in that same setting—addition of everolimus increased median PFS from 5.78 to 7.00 months (*P* = 0.0067), although the toxicity of the combination was a relevant concern (André et al. 2014). That same group proceeded to explore whether a biomarker in the BOLERO-3 and BOLERO-1 trials could better select for patients who would eventually benefit from combined therapy. These data suggested that the patients who might benefit most from the combination are the ones with hyperactive PI3K pathway, either through activating mutations in *PIK3CA* or PTEN loss. However, *PIK3CA* mutational status as a marker for improved PFS in response to everolimus reached statistical significance only when both studies (BOLERO1 and BOLERO3) were analyzed jointly (Andre et al. 2016).

When we compared our trastuzumab-resistant patients with TCGA BC cohort, we found that *BRAF, EGFR* and *KIT* mutations were significantly (*P* < 0.0020) enriched in our patient population. In *BRAF*, we found one patient had a G469E mutation and two others had an I326T mutation. Both are missense mutations. G469E localizes to kinase domain and probably leads to hyperactivation of the kinase (Davies et al. 2002), but it has not been described in primary breast cancer before (Forbes et al. 2015). The I326T mutation was described in breast cancer cell lines but its functional effect is yet unknown (Davies et al. 2002; Hollestelle et al. 2007; Sabine et al. 2014). It is conceivable that activation of B-Raf contributes mechanistically to resistance to Her2 blockade by leading to activation of the MAPK/ERK signaling pathway independent of engagement of epidermal growth factor receptors.

In *KIT*, we found two mutations in three patients, both localizing to the kinase domain: E839K and V825A. The inactivating E839K mutation was described in cutaneous mastocytosis and polycythemia vera (Fontalba et al. 2006; Longley et al. 1999) and the V825A mutation, also not activating, had been described in sinonasal lymphoma (Hongyo et al. 2000). This leads us to hypothesize that resistance to Her2 blockade can develop through intracellular activation of multiple different pro-survival signals that do not rely on cell surface receptors.

Finally, the R108K and R677H mutations we found in *EGFR* were previously described in glioblastoma (Lee et al. 2006) and glioma (Forbes et al. 2015). Intracellular EGFR signaling shares many of its intermediaries with Her2 pathway and hence overactivation of EGFR, making Her2 input redundant, is conceivable and lapatinib, which inhibits not only HER2/neu but also EGFR, might lead to better outcomes in these cases (Clavarezza et al. 2016).

Our findings are consistent with the results of a larger study (Nik-Zainal et al. 2016) in which the mutational signature of ER-positive versus ER-negative tumors was different, even in Her2-positive BC. Hence, we speculate that trastuzumab-resistant ER+ Her2-positive BC acquires mutations that impair DNA damage repair while trastuzumab-resistant ER− Her2-positive tumors acquire mutations that enhance mitotic signaling.

## Limitations

DNA was only available for 22/47 patients enrolled in the combined phase I/II trial. This meant there is potential for selection bias, although we have no reason to suspect that it was the case. It also means that the study might not be powered to detect differences between any two groups, which might explain why *PI3KCA* mutations or PTEN loss were not prognostic (beta error). Archival material was analyzed, and fresh pre- or on-study biopsies were not available. Furthermore, samples were not microdissected before DNA extraction. The chip-based Oncoscan^TM^ method has its limitations in terms of sensitivity and specificity (Villegas-Ruiz et al. 2016) and next-generation deep genome sequencing is the de facto gold-standard for detecting genomic mutations.

## Conclusion

In conclusion, *PI3KCA* mutation or PTEN loss was not prognostic for response to the combination of everolimus and trastuzumab in our cohort. Our data shows that mutational burden in heavily treated trastuzumab-resistant Her2-positive metastatic breast cancer is highly variable and not directly correlated with outcome. Activation of the MAPK/ERK pathway through mutations in *EGFR, BRAF* or *KIT* may mediate resistance to trastuzumab.

## Methods

### Sample collection

Tumor biopsies from primary and metastatic sites were collected and formalin-fixed/paraffin-embedded before initiation of everolimus as described (Morrow et al. 2011b). DNA from 36/47 samples was available for us to analyze. Material was processed for Affymetrix OncoScan™ FFPE Express 2.0 Services assay. After exclusion of samples derived from metastatic sites, we submitted samples to quality control based on the amount of DNA (>75 ng per ample) and median-absolute pairwise difference (MAPD <0.60). Nine ovarian tissue controls were used for adequate quality control. After QC, 22 samples and all of 9 controls were retained for analysis.

### Clinical data

Progression-free survival, estrogen receptor status and PTEN presence or loss data were collected as part of the original study protocol as published (Morrow et al. 2011b). PTEN expression levels were based on IHC staining and were available for 18/22 patients. All data made available for our analysis was de-identified.

### Bioinformatics

For each SNP detected by a specific probe in the OncoScan™ assay, a somatic mutation score was calculated $(\text{score}i\,=\,\text{abs}(\text{x}i-\text{ }\!\!\mu\!\!\text{ controls})/\sigma \text{controls}\times \text{sqrt}\left( \text{MAPD}\ i \right),$ where x*i* is the contrast of sample *i*. All calls detected with a somatic mutation score >5.0 were considered true positives. Finally, synonymous SNV were excluded from the final analysis.

### Data analysis

Using the method described above, 41 somatic mutations in 26 genes were identified. We queried TCGA data from the Breast Invasive Carcinoma project (Ciriello et al. 2015) using cbioportal.org. We queried only mutations and not CNA or mRNA expression levels. Furthermore, we queried only the genes that were found to be mutated in our cohort. We then compared our cohort with the whole breast invasive carcinoma TCGA dataset (*n* = 1105) or with Her2-positive breast tumors only (*n* = 120). We also directly compared the Her2-positive subgroup (*n* = 120) with TCGA Her2-negative BC (*n* = 985). To analyze differences in gene status (mutated versus wild type) in these cohorts, Fisher’s exact test was used and *P* value was defined as significant if *P* < 0.0020 (0.05 divided by 26—Bonferroni correction for multiple comparisons, being 26 the number of genes included).

We used a log-rank (Mantel–Cox) test to compare survival curves and statistical significance was defined as *P* < 0.05 for this analysis. All data analysis was done using GraphPad Prism (version 6.01 for Windows, GraphPad Software, La Jolla California USA, http://www.graphpad.com).
